# Manufacturing Techniques and Surface Engineering of Polymer Based Nanoparticles for Targeted Drug Delivery to Cancer

**DOI:** 10.3390/nano6020026

**Published:** 2016-02-01

**Authors:** Yichao Wang, Puwang Li, Thao Truong-Dinh Tran, Juan Zhang, Lingxue Kong

**Affiliations:** 1 School of Electrical and Computer Engineering, RMIT University, Melbourne, VIC 3000, Australia; yichaowang@gmail.com; 2 Institute for Frontier Materials, Deakin University, Locked Bag 20000, Geelong, VIC 3220, Australia; puwangli@163.com (P.L.); truongd@deakin.edu.au (T.T.-D.T.); jane.zhang@deakin.edu.au (J.Z.); 3 Agricultural Product Processing Research Institute, Chinese Academy of Tropical Agricultural Sciences, Zhanjiang 524001, China; 4 Pharmaceutical Engineering Laboratory, Biomedical Engineering Department, International University, Vietnam National University, Ho Chi Minh City 70000, Vietnam

**Keywords:** nanoparticles, PLGA, chitosan, nano/microencapsulation, drug delivery, top-down fabrication techniques

## Abstract

The evolution of polymer based nanoparticles as a drug delivery carrier via pharmaceutical nano/microencapsulation has greatly promoted the development of nano- and micro-medicine in the past few decades. Poly(lactide-co-glycolide) (PLGA) and chitosan, which are biodegradable and biocompatible polymers, have been approved by both the Food & Drug Administration (FDA) and European Medicine Agency (EMA), making them ideal biomaterials that can be advanced from laboratory development to clinical oral and parental administrations. PLGA and chitosan encapsulated nanoparticles (NPs) have successfully been developed as new oral drug delivery systems with demonstrated high efficacy. This review aims to provide a comprehensive overview of the fabrication of PLGA and chitosan particulate systems using nano/microencapsulation methods, the current progress and the future outlooks of the nanoparticulate drug delivery systems. Especially, we focus on the formulations and nano/micro-encapsulation techniques using top-down techniques. It also addresses how the different phases including the organic and aqueous ones in the emulsion system interact with each other and subsequently influence the properties of the drug delivery system. Besides, surface modification strategies which can effectively engineer intrinsic physicochemical properties are summarised. Finally, future perspectives and potential directions of PLGA and chitosan nano/microencapsulated drug systems are outlined.

## 1. Introduction

Cancer refers to a disease characterised by the uncontrolled growth and spread of abnormal body cells [1]. Current treatments of cancers include surgery, chemotherapy and radiation therapy. Surgery and radiation therapy are mostly applied for local and non-metastatic ones. Chemotherapy is frequently used when cancer has spread throughout the body. Therefore, chemotherapy forms a main strategy for the treatment of cancer before and after surgery and radiation therapy. Conventional chemotherapy kills not only the cancerous cells but also the healthy ones, which results in very strong side-effects. This significantly hampers the maximum administration of chemotherapeutic drugs. Moreover, a short half life time and rapid plasma clearance necessitate the administration of a high concentration of drugs, which is not economic and often leads to unexpected toxicity issues [2]. NPs are tailor-made drug delivery carriers that are capable of transporting high doses of chemotherapeutic agents into the cancerous cells while sparing healthy cells. NPs have shown great promise in changing the face of oncology as they have the ability of cell-specific targeting and sustained drug release [3], which overcomes the limitations of traditional chemotherapy.

Polymer based nanoparticle drug delivery carriers have been extensively studied in the pharmaceutical field [4]. Poly(lactide-co-glycolide) (PLGA) and chitosan are two of the most typical biodegradable and biocompatible polymers and have been approved by both the Food & Drug Administration and European Medicine Agency, signifying their transition from the laboratory to clinical oral and parental administration. Commonly used methods for synthesising PLGA nanoparticles (NPs) consist of top-down and bottom-up methods. The top-down techniques usually employ as-prepared polymers to synthesize NPs [5], such as emulsion diffusion, salting out, nanoprecipitation method, and emulsion evaporation. The bottom-up methods normally start from a monomer [5]. The precipitation polymerization, emulsion and microemulsion polymerization and interfacial polymerization are the specific forms. In general, all of these methods are composed of two major steps. They share the first step which is to prepare an emulsified system. The NPs are hardened and formed in the second step, which is varied for different top-down techniques mentioned above.

As PLGA is one kind of polyesters, it does not have sufficient functional groups that could enhance its potential applications. Extensive work has been carried out to functionalise PLGA surface, in order to enhance the performance of PLGA-based drug delivery system. Here, the improved hydrophilicity, chitosan functionalization, targeting and pH sensitive coating are summarised.

This review details the nano/microencapsulation techniques and surface functionalization strategies of polymer based NPs for cancer therapy.

## 2. Microencapsulation Techniques

Over the past decade, there has been increasing interest in using polymer based NPs for cancer therapy. The techniques for the preparation of PLGA and chitosan NPs are summarized in the following sections.

### 2.1. Microencapsulation of PLGA Nanoparticles

#### 2.1.1. Emulsion Diffusion Method

**Figure 1:**
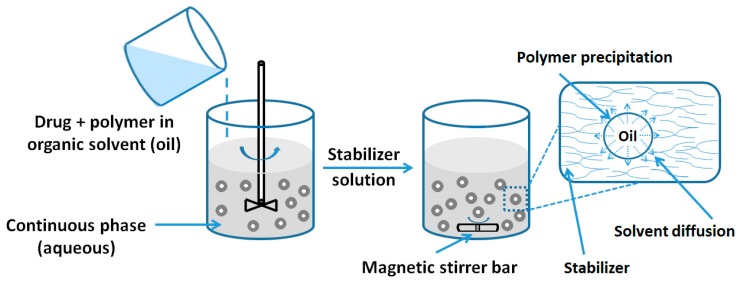
Preparation of nanocapsules by emulsion diffusion method.

There are single emulsion and double emulsion systems in this fabrication method. Single emulsion encapsulation method is conducted for the formulation of oil soluble (hydrophobic) substances [6], while double emulsion is adopted by entrapment of hydrophilic chemicals [7,8]. The schematic of the emulsion diffusion fabrication processes is presented in Figure 1.

One of the key requirements of the emulsion diffusion method is the selection of an organic phase (oil phase) containing PLGA solution which must be partially miscible in aqueous phase. The most important fabrication step is solvent diffusion, in which the organic phase diffuses from the oil phase to outer water phase and the formed particles become hardened. The selection of the surfactants in the outer water phase is also crucial to the successful fabrication. Different kinds of surfactants, such as non-ionic surfactant polyvinyl alcohol (PVA) [9], anionic surfactant sodium dodecyl sulphate (SDS) [5] and cationic surfactant didodecyl dimethyl ammonium bromide (DMAB) [10], are commonly applied based on emulsion systems. Different surfactants can induce particles in different sizes [11]. Budhian *et al.* [12] reported that when DMAB was used as surfactant for the fabrication of PLGA NPs, smaller particles were fabricated than the ones prepared by using PVA as the surfactant. Another popular stabilizer for the fabrication of PLGA NPs is amphiphilic d-α-tocopheryl polyethylene glycol 100 succinate Vitamin E (TPGS) [13,14] as TPGS has very high emulsion efficiency and can enhance the cellular adhesion. The amount of TPGS used as surfactant usually can be as low as 0.015% (*w/v*).

The amount of surfactant used has an effect on the properties of the NPs. Low concentration of surfactants usually leads to a high polydispersity and particle aggregation [15]. However, if excessive surfactants are used, the drug loading will decrease due to a strong interaction between the drugs and surfactants. Therefore, the suitable concentration of surfactant is the key to successful fabrication. Another method to form the mono-dispersed emulsion is using the probe sonicator to impose high energy in the formed emulsion [16]. The selection of specific sonicator mode, time and power is essential to the formation of emulsions.

#### 2.1.2. Salting out Method

**Figure 2:**
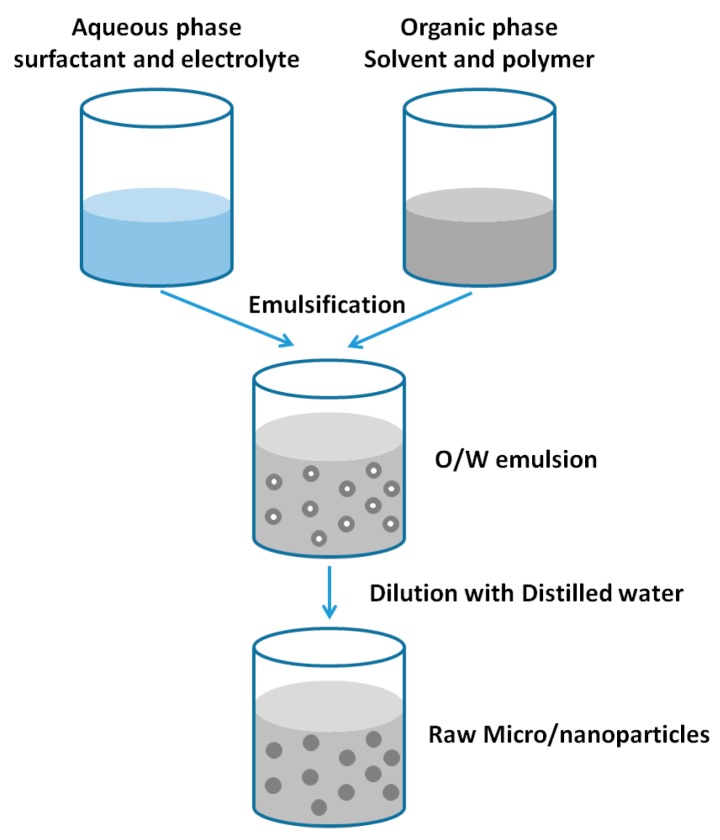
Preparation of nanoparticles (NPs) by salting out method.

Salting out is another method for the fabrication of PLGA NPs. Firstly, the PLGA is dissolved into the organic solutions (oil phase) which are usually water-miscible. Typical solvents are tetrahydrofuran (THF) and acetone. The aqueous phase consists of the surfactant and saturated solution of electrolyte. The electrolytes should not be soluble in the organic solvent. Typically, the most commonly used salts are magnesium chloride hexahydrate with a concentration of 60% (*w/w*) [17] or magnesium acetate tetrahydrate which is normally used with a ratio of 1:3 (polymer to salt) [18]. The oil phase is emulsified in an aqueous phase, under strong shearing force by overhead mechanical stirrer. The obvious difference between the emulsion diffusion method and salting out method is that there is no solvent diffusion step for the latter one due to the existence of salts. In order to decrease the ionic strength in the electrolyte, the distilled water is added into the formed O/W emulsion under magnetic stirrer. At the same time, the hydrophilic organic solvents migrate from the oil phase to aqueous phase, which results in the formation of the NPs. Finally, the salting out agent is eliminated by centrifugation and the samples are purified. The schematic of the salting out processes is presented in Figure 2.

#### 2.1.3. Nanoprecipitation Method

**Figure 3:**
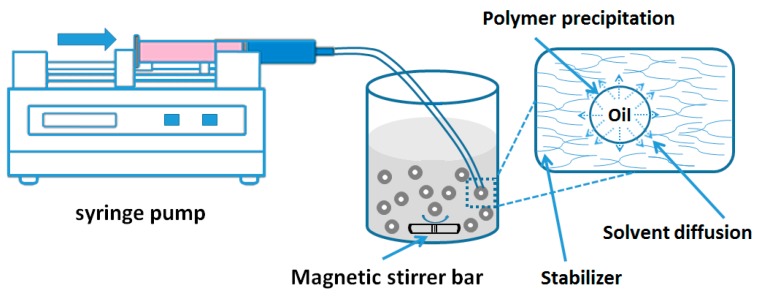
Preparation of NPs by nanoprecipitation method.

Nanoprecipitation is also called solvent displacement or interfacial deposition method, which was first developed and introduced by Fessi’s group [19]. The principle of this fabrication method is known as Marangoni effect [20]. In the nanoprecipitation method, the nanoparticles are obtained in the colloidal suspension when the oil phase is slowly added to aqueous phase under moderate stirring (Figure 3). Formation of the NPs is instantaneous and needs only one step so it has the advantage of rapid and easy operation. The key parameters in the fabrication procedure have great influence on the nanoprecipitation method, such as organic phase injection rate, aqueous phase agitation rate and the oil phase/aqueous phase ratio [8]. Particle sizes of very narrow distribution can be synthesised because of the absence of shearing stress. This method is used mostly for hydrophobic drug entrapment [21], but it is also employed sometimes to incorporate hydrophilic drugs [22]. Polymer and drug are dissolved in a water miscible organic solvent, for example, acetone or methanol. The solution is then added into an aqueous solution which contains surfactant in a drop-wise manner. Through rapid solvent diffusion, the NPs are formed immediately. After that, the solvents are removed under reduced pressure.

#### 2.1.4. Emulsion Evaporation Method

**Figure 4:**
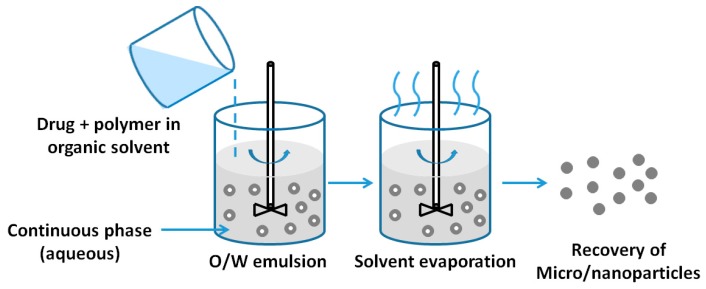
Preparation of NPs by emulsion evaporation method.

Emulsion evaporation has been used for a long time to form polymeric NPs from as-prepared polymers [8,23]. The method is based on the emulsification of polymer organic solution into a water phase, followed by organic solvent evaporation. The polymer is first dissolved in a suitable solvent (e.g., ethyl acetate, chloroform, or methylene chloride). The organic phase is poured into the continuous phase (aqueous phase) in which a surfactant is dissolved to impart stability to the emulsion [24]. Emulsification is carried out under high-shear force to reduce the size of the emulsion droplet. This process will largely determine the final particle size. After the formation of emulsification, the system evaporates the organic solvent under vacuum, which leads to polymer precipitation and nanoparticle formation [23]. The schematic of the emulsion evaporation processes is illustrated in Figure 4.

From this section, the fabrication methods including emulsion diffusion, salting out, nanoprecipitation and emulsion evaporation for PLGA oral drug delivery carriers are described. Drugs loaded PLGA NPs can be obtained by using these techniques. Although the PLGA based drug delivery carriers can obtain sustained and prolonged drug release profiles through the degradation of PLGA matrix, the systems have difficulties in achieving the functionalization, and thus their usage is always limited. In order to maximize the efficacy of oral drug delivery carriers, multi-functions such as drug targeting and pH-sensitivity have to be achieved. Therefore, different approaches for modifications of PLGA drug delivery carriers are described in the next section.

### 2.2. Microencapsulation Techniques for Chitosan

#### 2.2.1. Ionic Gelation

**Figure 5:**
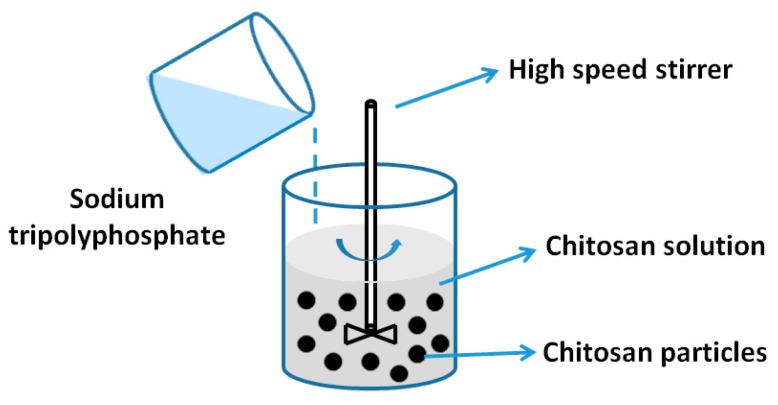
Preparation of chitosan NPs by ion gelation technology.

The formulation of chitosan NPs by ionic crosslinking technology (Figure 5) is based on the formation of complexation between positively charged amine group of chitosan and negatively charged polyanion such as tripolyphosphate (TPP) [25]. The process is simple and mild, and the entire preparation process can be conducted in aqueous condition without the use of any organic solvent. Due to this unique characteristic, chitosan NPs have been widely explored in pharmaceutical applications. In this method, cationic solution of chitosan was previously obtained by dissolving in diluted acetic acid, and anionic solution of TPP was obtained by dissolving it in distilled water. Then, TPP solution was added drop-wise into the cationic solution of chitosan. NPs were formed instantly under mechanical stirring at room temperature. Physiochemical properties of the resultant NPs such as the particle size and surface charge could be modulated by varying the concentration of chitosan and crosslinking agent, and the pH value of the solution [26].

Gan and Wang [27] prepared bovine serum albumin (BSA)-loaded chitosan NPs based on simple and mild ionic gelation of chitosan or its derivative with TPP. The preparation parameters that influenced the preparation of NPs, including chitosan concentration, chitosan molecular weight, BSA initial concentration, and chitosan/TPP mass ratio, were examined. Chitosan and its derivatives were also used to prepare NPs [28] and study the influence of TPP concentration and the chemical modification of chitosan on the particle size. It was observed that the particles’ size increased with TPP concentration and the particle size of the quaternized chitosan NPs is larger than that of NPs made from unmodified chitosan. Higher encapsulation efficiency and loading capacity were also achieved by quaternized chitosan NPs.

#### 2.2.2. Reverse Micellar Method

**Figure 6:**
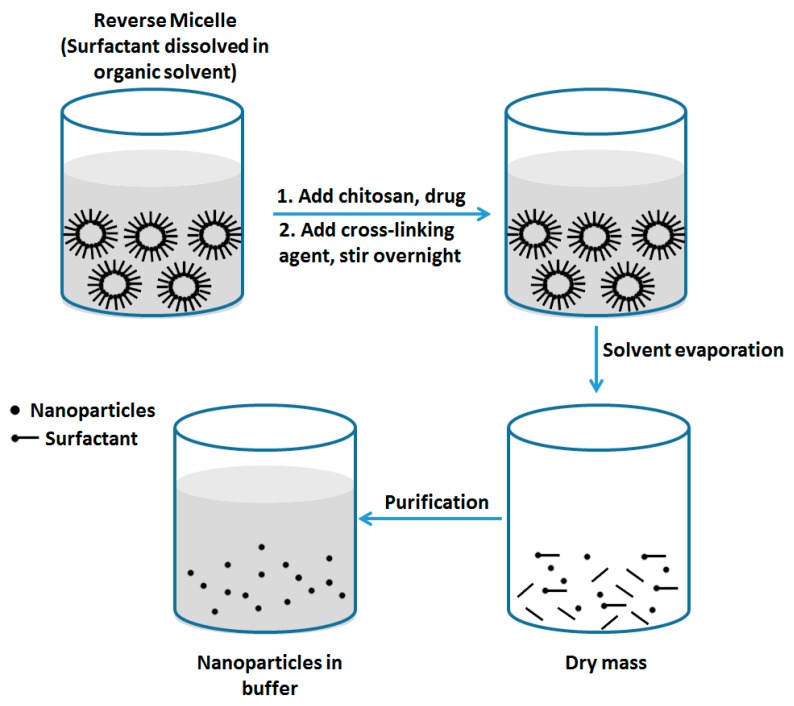
Preparation of chitosan NPs by reverse micellar method.

The preparation of chitosan NPs by reverse micellar method is based on the formation of NPs in the aqueous core of reverse micellar droplets and subsequently crosslinking with glutaraldehyde (Figure 6) [29]. In this method, a surfactant was dissolved into an organic solvent to form reverse micelles. To do this, aqueous chitosan solution was added under continuous stirring to avoid turbidity. A crosslinking agent was added to this transparent solution under constant stirring. The system was maintained stirring overnight to accomplish the cross-linking process and to ensure that the free amine group of chitosan conjugated with glutaraldehyde. The organic solvent was then removed by evaporation under low pressure. The yields obtained were the cross-linked chitosan NP and excess surfactant. The excess surfactant was removed by precipitate with suitable salt and then the precipitant was removed by centrifugation. Final NPs suspension was dialyzed before lyophilyzation. Chitosan NPs prepared by this technology were less than 100 nm and were highly mono-dispersed. The size of particle can be modulated by varying the amount of glutaraldehyde which altered the degree of crosslinking. Nevertheless, there are some limitations with this technology including the use of organic solvent, the time consuming preparation process and the complex washing step.

#### 2.2.3. Emulsification Solvent Diffusion

The preparation of chitosan NPs by emulsification solvent diffusion technology relies on the crosslinking between the reactive functional amine groups of chitosan and aldehyde groups, and the partial miscibility of an organic solvent with water. Glutaraldehyde and formaldehyde were extensively used as cross-linkers for the preparation of chitosan particles. Recently, vanillin was also used as cross-linkers for the preparation of chitosan particles to avoid the toxicity of glutaraldehyde and formaldehyde. Vanillin obtained from vanilla pods is an important flavouring agent which is widely used in industries such as food, beverage and cosmetic [30]. In this method, an organic phase was injected into chitosan solution containing stabilizing agent under high shearing force, followed by homogenization process under high pressure [31,32]. After that, a large amount of water was poured into the system to overcome the organic solvent miscibility in water. Polymer precipitation occurs upon the diffusion of organic solvent into water, which subsequently leads to the formation of NPs. This method is suitable for hydrophobic drug and a high percentage of drug entrapment could be achieved. However, it requires the use of organic solvents and high shearing forces.

#### 2.2.4. Spray-Drying Method

**Figure 7:**
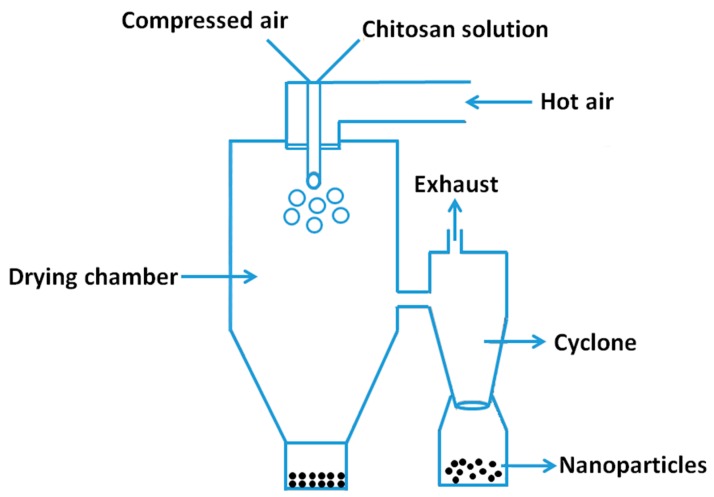
Preparation of chitosan particulate systems by spray drying method.

Drug loaded powders, granules or agglomerates can be prepared by spray-drying method. Mixture solution of drug and excipient was atomized into small droplets and blown into a chamber filled with hot air. Drug loaded particles were obtained upon the drying of droplets. The preparation of drug loaded NPs by spray-drying method was shown in Figure 7. Firstly, chitosan solution was made by dissolving it in diluted aqueous acetic acid solution, then, drugs were suspended or dissolved in the chitosan solution. After that, a selected cross-linking agent was dropped into the mixture solution of chitosan and drugs. This solution was then atomized into a chamber with a stream of hot air. Small droplets were formed upon the atomization and the formation of flowing particles with evaporation of solvent. The particle size of the final products could be modulated by varying the preparation parameters, such as atomization pressure, spray flow rate, the nozzle size, inlet air temperature, and extent of crosslinking. This technique is suitable for the encapsulation of either water soluble and water-insoluble heat-resistant or heat-resistant and heat-sensitive drugs.

#### 2.2.5. Coacervation/Precipitation

**Figure 8:**
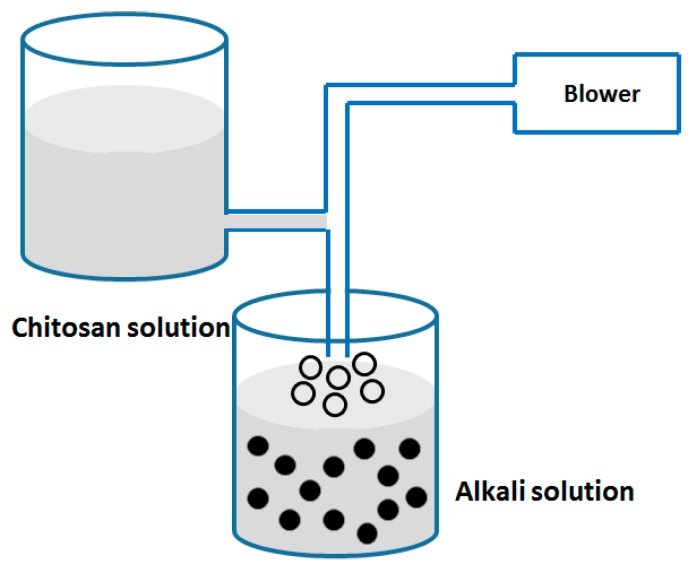
Preparation of chitosan NPs by coacervation/precipitation method.

As well established, chitosan CS is soluble in solution with pH value less than 6.5, but it precipitates/coacervates in alkaline solution (pH > 6.5). The preparation of NPs using coacervation/ precipitation method is based on this unique property. As shown in Figure 8, the chitosan NPs were prepared by injecting chitosan solution into basic organic solvent (sodium hydroxide, NaOH methanol or ethanediamine) via a compressed air nozzle. The NPs were collected by filtration or centrifugation and washed repeatedly with water. The size of the chitosan NPs could be modulated by varying compressed air pressure or spray-nozzle diameter. Crosslinking agent is used to harden the particles in order to control the drug release profile.

## 3. Modification Techniques

Although PLGA and chitosan are biodegradable and biocompatible materials and have the potential to be used in many areas, their applications are often limited due to their lack of suitable functional groups [33]. Therefore, a variety of attempts have been made to functionalize PLGA, chitosan and their NPs, including the improvement of hydrophilicity of polymer and the conjugation with targeting ligand for the increase of targeting efficiency [34,35]. Some of the modification techniques for the polymer and polymer based drug delivery NPs were described in the following sections.

### 3.1. Techniques for the Modification PLGA or PLGA Particles

#### 3.1.1. Improving Hydrophilicity

PLGA is a naturally hydrophobic material, like most of the biodegradable polyesters. Its hydrophobic index is dependent on the ratio between the amount of two monomers lactic acid (LA) and glycolic acid (GA).

Poor hydrophilicity always limits the practical drug formulations of PLGA, especially for the entrapment of hydrophilic drugs. Also, the hydrophobic drug carriers are recognized as a foreign substance by the body. When administered, the drug carriers with hydrophobic surface are surrounded by mononuclear phagocytic system (MPS), which can absorb the carriers, especially in the liver. Therefore, one of the purposes for the surface modification with hydrophilic components is to make the carriers unrecognizable by the MPS.

To enhance the hydrophilicity and other physicochemical properties, poly(ethylene glycol) (PEG) has been conjugated onto PLGA. PEG is a biocompatible, non-toxic and water soluble polymer. Block copolymers composed of PLGA segment and PEG segment have attracted much interest in recent years due to their biodegradability, biocompatibility and tailer-made properties [36]. A number of drug formulations including nanoparticles were achieved by using PLGA-PEG and PLGA-mPEG [37]. It was also found that the dissolution rate of PLGA-PEG copolymers is much higher than that of unmodified PLGA due to enhanced hydrophilicity [38]. Natural polysaccharide chitin was investigated to form chitin/PLGA blends [39]. Chitin is more hydrophilic than PLGA. There are β-glycosidic bonds between d-acetylglucosamine units where the cleavage leads to the chitin degradation. It was reported that the drug release from the chitin/PLGA blending was influenced by the hydrophilicity enhancement [39]. The d-α-tocopheryl polyethylene glycol 1000 succinate (TPGS) has been used as an emulsifier and segment of drug delivery vehicles for many years [40]. The PLGA/TPGS NPs were found to have a high encapsulation efficiency [41].

#### 3.1.2. Chitosan Functionalization

**Figure 9:**
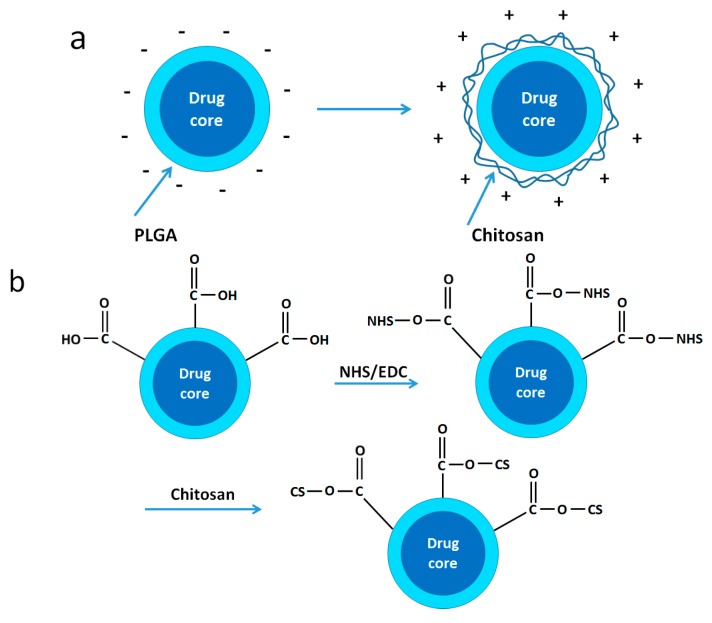
Schematic maps of chitosan modified poly(lactide-co-glycolide) nanoparticles (PLGA NPs) by (**a**) physical adsorption method and (**b**) chemical binding method. Reproduced with permission of [45]. Copyright Springer, 2016.

In order to improve the functionality of PLGA surface, a conjugate can be synthesized by grafting chitosan on PLGA via the amino groups (Figure 9a,b). Chitosan functionalization on the PLGA surface explores the broader utilization of this polymer. The surface modification of chitosan can also increase the particle surface zeta potential, which is beneficial for the *in vitro* cytotoxic effects. Chitosan is a natural cationic polymer which has been widely used in the pharmaceutical area. It has a very good biocompatibility, biodegradability and nontoxicity. Therefore, using chitosan and its derivatives to prepare NPs or coated onto nanoparticle surface has been widely investigated in recent years [42]. The surfaces of chitosan NPs have a relatively high zeta potential, which adheres strongly to the cells as cancer cells generally have a negative charge on their surface [43]. As NH_2_ groups exist in each unit of chitosan, PLGA is grafted onto the chain randomly. Therefore, the crystallinity of chitosan is largely affected. Chitosan also acts as the bridge to form PLGA based triblock polymers. Alginate-chitosan-PLGA composite containing Hepatitis B vaccine was prepared by Liang’s group [44]. This system showed improvement in encapsulated protein stability, drug release and antibody levels [44].

#### 3.1.3. Targeting Functionalization

**Figure 10:**
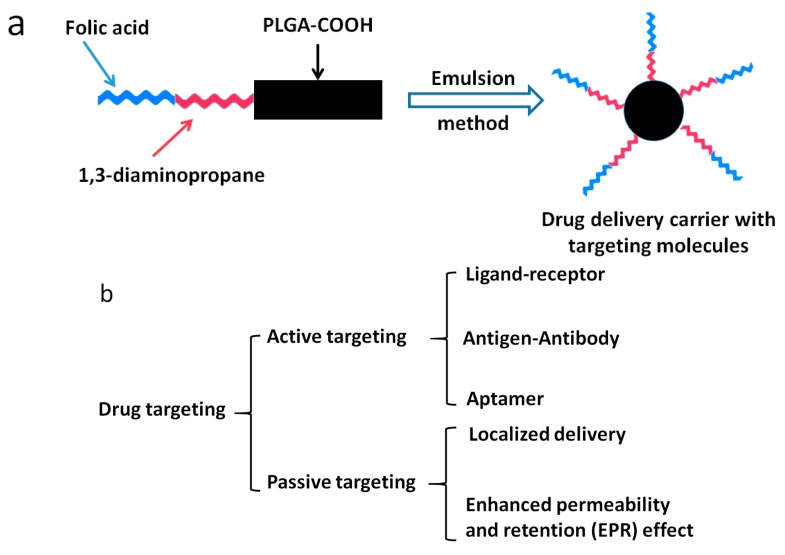
(**a**) Formation of the PLGA-1, 3-diaminopropane-folic acid targeting drug delivery system. (**b**) Classification of targeting drug delivery system.

The concept of selective drug delivery system was introduced by Paul Erhlich 100 years ago [46]. The “Magic Bullet” proposed has great affinity and specificity to the cells, tissue and organs in the human body [47]. Targeting functionalization of PLGA surfaces enables the drugs to be delivered to the designated area and to specifically target the cancerous cells (Figure 10a). Therefore, the drug carriers reach the cancer sites with specificity and affinity, while having less side-effects on the healthy tissues. The transportation of the drugs to the designated areas can be mainly achieved in two ways: active and passive targeting (Figure 10b), and each can be divided into several sub-categories.

#### 3.1.4. pH Sensitive Coating

A pH sensitive coating can protect the drug loaded PLGA NPs from being released in the oral delivery route. As many drug delivery carriers are administered orally, it would be more promising if the drug carriers are pH-sensitive as the gastrointestinal tract which the drug delivery carriers pass through is an environment with varying pH values. The properties of water-insolubility at low pH and water-solubility at high pH polymers have been of great interest in recent years. The release rate and the timing of the drugs to be released can be controlled and triggered by the pH values of the environments. However, the drug release mechanisms from the core-coated particles are not fully understood yet [48]. There are many complicated processes, such as water imbibition, drug diffusion and dissolution through the intact nanoparticles. For example, it was reported that the release of chemotherapeutic drug phenylpropanolamine hydrochloride from ethylcellulose coated particles was manipulated not only by the drug dissolution but also an osmotic effect [49]. Also, the cracks could be derived from hydrostatic pressure in coated particles for different drug delivery systems [50]. Eudragit S100 is another popular pH sensitive polymer and widely used for coating the PLGA NPs due to the existence of carboxylic acid group which make the polymer pH sensitive and insoluble under pH 7. There are carboxyl and ester groups in the polymer structure of Eudragit S100 and the ratio is 1:2.

#### 3.1.5. Plasma Treatment

**Figure 11:**
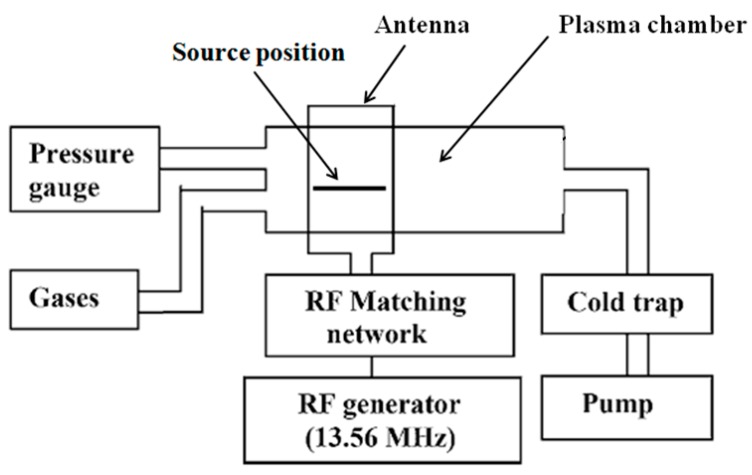
Schematic diagram of treatment chamber for gas plasma polymerization.

Modification by plasma technology can be divided into two categories: one is gas plasma treatment and the other is plasma polymerization. Plasma treatment is very convenient for material surface modification [51]. It can treat biomaterials with complex shapes and surfaces. As this technology can very easily introduce the reactive groups or chains onto the material surfaces, it is commonly applied for the enhancement of cell affinity [51]. For gas plasma treatment, different gases, such as oxygen, ammonia, combination of nitrogen and hydrogen, were used for immobilization of different functional groups, including amine, hydrogen and carboxylic groups. The schematic diagram of treatment chamber for gas plasma polymerization is presented in Figure 11.

The modification of polymer surfaces by plasma polymerization is substrate independent. Plasma polymerization involves substrate fragmentation and deposition of organic monomers. Due to the deficiency of suitable functional groups on their surface, conventional PLGA NPs lack the possibility of specific targeting or biominmetic purposes. The surface properties of NPs are important for the performance of NPs *in vivo*. Plasma modifications will greatly improve the effectiveness of NPs’ delivery system and cell affinity. Lee *et al.* investigated the PLGA surface modification by plasma treatment, in order to induce the cell affinity on the polymer surface [52]. The tests showed that cell proliferation was significantly enhanced in the human dermal fibroblast (HDF) attachment after six days of incubation due to improved hydrophilicity of PLGA film by plasma treatment. Oxygen plasma could also be used for PLGA surface modification for the immobilization of laminin [53] and the proportion of laminin incorporated on PLGA surface by plasma treatment was much higher than that by conventional chemical methods. Also, plasma treatment can make the PLGA surface more hydrophilic. Hasirci *et al*. [54] applied oxygen plasma treatment to modify PLGA surface and found the PLGA water contact angle decreased from 67° to 38° after the treatment.

Different from the gas plasma treatment, plasma polymerization coats the organic monomer onto the substrate rather than covalently binding various functional groups onto the polymer surface. Currently, one of the commonly used monomers for the PLGA plasma polymerization is monomer allylamine. In a study by Modo [55], polymerized allylamine treated PLGA scaffold particles as a structural support for the neural cells in the brain. The results indicated that the plasma treated scaffolds ensured the cell grafting and improved the recovery of damaged brain cells. Heptylamine is another monomer for plasma polymerization as it mainly incorporates the amine groups on the PLGA surface and has been confirmed to be a useful medium for subsequent biomolecule grafting, and three-dimensional functionalization could be achieved [56].

### 3.2. Techniques for Modification of Chitosan and Chitosan Particles

#### 3.2.1. *N*-Trimethyl Chitosan Chloride (TMC)

It has been proved that TMC could effectively increase the permeation of hydrophilic macromolecular drugs across the mucosal epithelia by opening the tight junctions. TMC is a water soluble chitosan derivative, which can be prepared by reductive methylation chitosan under basic preparation conditions and proper reaction temperatures [57]. The quaternization degree of primary amino group and methylation of 3- and 6-hydroxyl groups were affected by the number of methylation processes and the base solution. TMC is able to open the tight junctions of intestinal epithelial cells and improve the permeation of hydrophilic macromolecular drugs across mucosal epithelia [58,59]. The charge density of TMC is an important factor determining its potential use as an absorption enhancer across intestinal epithelia. The permeability of TMC was assayed in intestinal epithelial cells. It was observed that TMC with a high degree of quaternization showed more efficient permeation as compared with TMC with a low degree of quaternization [57].

#### 3.2.2. Poly(Ethylene Glycol) Modified Chitosan

As mentioned previously, the application of chitosan in pharmaceutical and biomedical fields is often limited by its poor solubility in water and organic solvent. To circumvent this limitation, attempts have also been made to graft hydrophilic groups into the function groups of chitosan. Among them, poly(ethylene glycols) (PEG) was extensively investigated. PEG is an amphipathic polymer which is widely used in chemical modification of artificial and natural polymers for pharmaceutical applications. It was reported that colloidal particles coated by PEG could avoid being recognized as foreign objects and thus could avoid being eliminated by phagocytosis [60,61]. It is also demonstrated that PEG can reduce immunoreactions of the body against polymers [62].

PEG-grafted chitosan was synthesized by *N*-substitution of triphenylmethyl chitosan with methoxy poly(ethylene glycol) iodide in organic solvent [63]. Grafted copolymers were collected by removing the triphenylmethyl groups [62]. The resultant copolymers displayed very good solubility in water over a wide range of pH value. To increase the solubility and biocompatibility of modified chitosan, Zhu *et al.* [64] synthesised PEG modified chitosan by free radical polymerization of chitosan and *N*-trimethylaminoethylmethacrylate chloride. This chemical modification by PEG could reduce the haemolytic to a half. PEG modified chitosan can also be prepared by reducing the amination of chitosan using PEG-aldehyde [65].

#### 3.2.3. Galactosylated Chitosan (GC)

**Figure 12:**
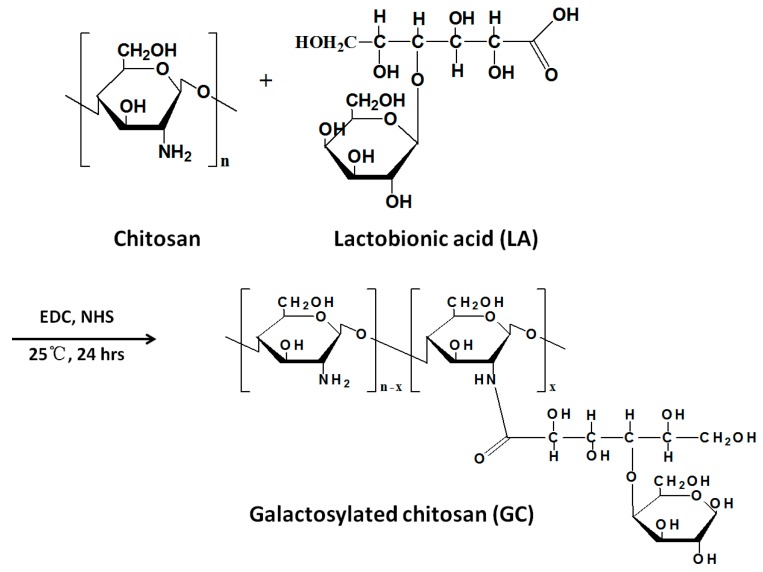
Preparation of galactosylated chitosan (GC).

GC can be prepared by substituting the amine group of chitosan with galactose group (Figure 12). Although only part of the amino groups in chitosan was substituted by galactosylate, the acyl reaction significantly changed the physicochemical properties such as the crystallinity, solubility, and stability of its resultant derivatives. Chitosan and GC particles were synthesized by physical precipitation and coacervation techniques, respectively. Both chitosan and GC particles were spherical in shape with an average diameter of 0.54 and 1.05 μm. They were both positively charged with average zeta potential of +17 and +15 mV, respectively. Due to their novel properties, GC particles could be used for passively and actively targeting drugs to liver [66]. The coated particles showed delayed and decreased burst release *in vitro*. Norcantharidin (NCTD) incorporated GC NPs were prepared by ionic crosslinking between the molecules of the NCTD and GC by Wang *et al.* [67]. The average particle size of the NCTD-GC NPs was 118.68 ± 3.37 nm, drug encapsulation efficiency and loading capacity were 57.92% ± 0.4% and 10.38% ± 0.06%, respectively. NCTD-GC NPs displayed improved compatibility with hepatoma cells and increased cytotoxicity against hepatocellular carcinoma cells.

#### 3.2.4. *N*-(2-hydroxyl) Propyl-3-Trimethyl Ammonium Chitosan Chloride (HTCC)

**Figure 13:**
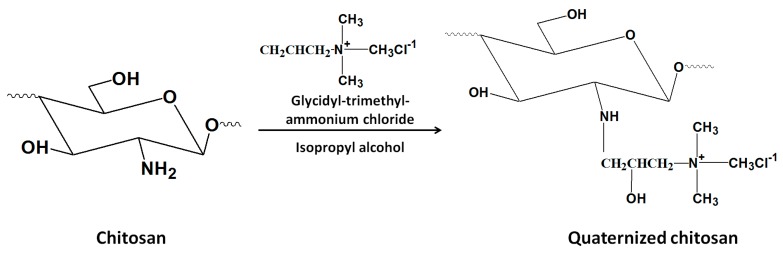
Preparation of *N*-(2-hydroxyl)propyl-3-trimethylammonium chitosan chloride (HTCC).

HTCC is one of the water soluble chitosan derivatives that can be synthesized by the chemical reaction of chitosan and glycidyl-trimethyl-ammonium chloride (GTMAC) in isopropyl alcohol (Figure 13). The resultant quaternized chitosan displayed excellent mucoadhesive and enhanced permeability properties. Therefore, it has great potential to be used as absorption enhancer [68]. HTCC NPs with a particle size of 110–180 nm could be prepared by ionic crosslinking of HTCC with sodium tripolyphosphate (TPP), and an encapsulation efficiency of 90% could be achieved by using bovine serum albumin (BSA) as model of protein drug [28].

#### 3.2.5. *O*-(2-Hydroxyl) Propyl-Trimethyl Ammonium Chitosan Chloride (O-HTCC)

**Figure 14:**
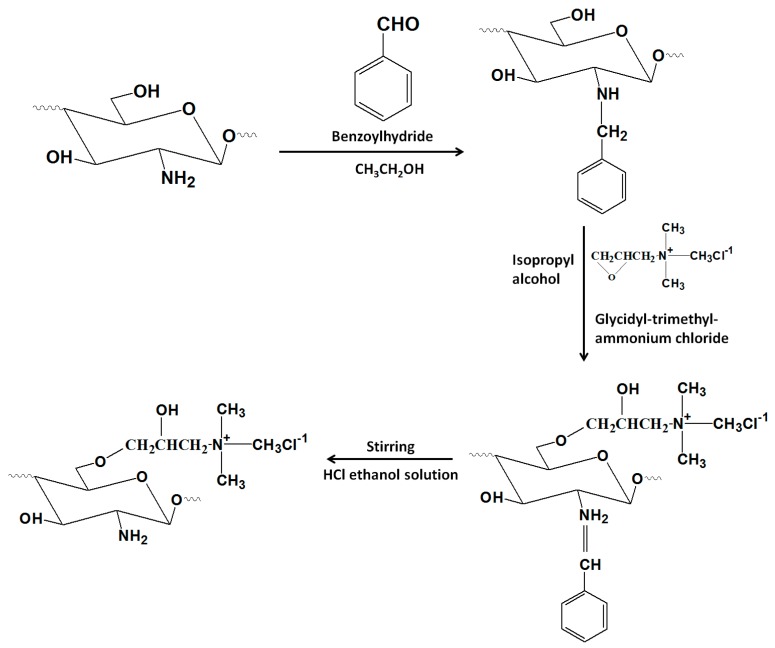
Schematic representative of preparation of *O*-(2-hydroxyl)propyl-3-trimethylammonium chitosan chloride (O-HTCC).

As shown in Figure 14, the preparation of O-HTCC consists of three steps: (a) the protection of functional groups (-NH_2_) in the C-2 position of chitosan with benzoyl hydride; (b) the interaction between glycidyl-trimethylammonium chloride (GTMAC) and *N*-benzylidene chitosan; (c) the removal of benzoylidene groups from the *O*-quaternary aminonium-*N*-benzylidene chitosan. O-HTCC was purified by dialysis technology and dried in vacuum freeze-drier [28]. The important application of O-HTCC was assayed by taking bovine serum albumin (BSA) as a model drug. Improved encapsulation efficiency and loading capacity was achieved by O-HTCC NPs as compared with chitosan NPs.

#### 3.2.6. Thiolated Chitosan

**Figure 15:**
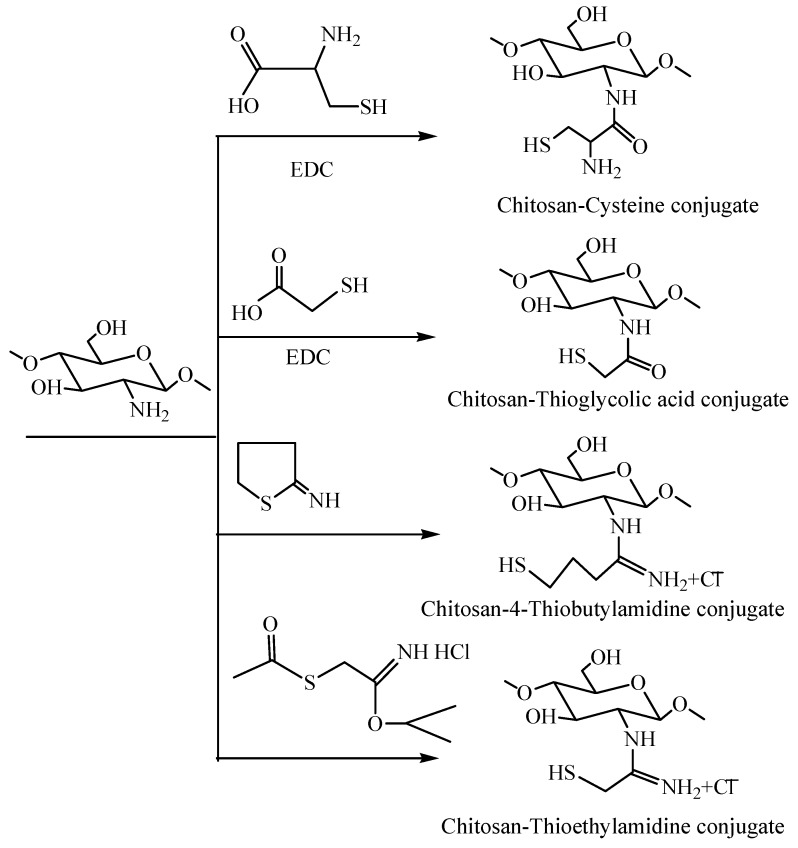
Preparation of thiolated chitosan.

As shown in Figure 15, the covalent attachment of sulfhydryl bearing reagents with the amino groups of chitosan (C-2) leads to the formulation of thiolated chitosan [69]. The chemical modification of chitosan introduced novel and improved properties of thiomers, making them mucoadhesive, permeation-enhancing, cohesive, and biodegradable, in addition to introducing enzyme inhibitory properties [69]. Thiolated polymers have been exploited to improve the mucoadhesive properties of drug delivery systems [70,71,72,73] with the retention time in the gastrointestinal tract being significantly prolonged and the drug absorption in lumen being markedly improved [74].

#### 3.2.7. Modification of Chitosan NPs with Targeting Agent

Active targeting can be achieved by functionalizing chitosan NPs with ligand such as antibodies, peptides, nucleic acid aptamers, carbohydrates, and small molecules (like folate acid [34,75,76] and hyaluronic acid). The presence of many function groups such as the amino groups and hydroxyl groups on the surface of chitosan NPs makes it amenable to various modifications. The conjugated targeting ligands to NPs have resulted in more efficient targeted therapeutics. For example, hyaluronic acid (HA) coupled chitosan NPs were prepared by covalent coupling of carboxyl group of HA with free amino group of chitosan present on the surface of NPs using EDC as coupling agent. HA coupled NPs showed a significantly higher uptake by cancer cells as compared to uncoupled NPs [77].

## 4. Conclusions

Over the past decade, there has been an increasing interest in using polymer based NPs for cancer therapy. The development of polymer based NPs that can deliver drugs directly to cancer cells at a sustained and controlled rate may provide better efficacy and lower toxicity for the treatment of cancer. We review the technologies for the fabrication of polymer based drug delivery systems for targeting cancer, and the surface engineering technologies for PLGA, chitosan, and their NPs. More efficient surface engineering technologies will result in the development of advanced polymer based drug delivery systems for targeted drug delivery to cancer cells.
